# Editorial: Cognitive Behavior Therapy and Executive Function in Neurodegenerative Disorders, Psychiatric Disorders, and Injury Research

**DOI:** 10.3389/fpsyt.2021.780748

**Published:** 2021-11-02

**Authors:** Jonathan M. Barron, Eric A. Storch

**Affiliations:** Department of Psychiatry and Behavioral Sciences, Baylor College of Medicine, Houston, TX, United States

**Keywords:** cognitive behavior therapy (CBT), executive function, diabetes, insomnia, depression

There have been numerous advances in healthcare in recent years, both technologically in the form of telehealth and internet-based innovations for patient connectivity; and medically in the form of cardiovascular, neurodegenerative, and oncological treatments. One area of healthcare that has lagged in terms of innovative treatments are those concerning mental and behavioral (i.e., how cognition and behavior impact mental/physical health) health. In the realm of psychosocial interventions, cognitive behavioral therapy (CBT) has a robust track record for treating primary psychiatric conditions such as various anxiety and mood disorders. There are numerous validated CBT protocols. While the components of each differ as a function of focus and disease state (e.g., CBT for obsessive-compulsive disorder involves a technique called exposure and response prevention; CBT for depression includes cognitive restructuring and behavioral activation), CBT is a structured form of psychotherapy that addresses cognitive and behavioral patterns of functioning that are theoretically linked to psychopathology and/or problematic functioning (e.g., poor adherence to medical regimens).

The application of CBT to primarily non-psychiatric conditions has received support in numerous studies for certain disease states, yet immediate uptake in the real-world remains limited and further data are needed in other understudied domains. As the number of unique behavioral therapy modalities have increased and been adopted for various permutations of psychiatric disorders, it has also been welcoming to see psychosocial interventions being applied in cases that previously had not received consideration. Indeed, this special issue of Frontiers in Psychiatry–Cognitive Behavior Therapy and Executive Function in Neurodegenerative Disorders, Psychiatric Disorders, and Injury Research—highlights a number of cutting edge efforts to advance the field. As such, the purpose of this editorial is to highlight papers in this issue in which CBT and other psychological interventions have been found useful or generated further scholarly interest.

One area of interest has been the utility of psychosocial interventions for individuals with non-psychiatric chronic medical conditions. Regarding diabetes mellitus, a chronic condition affecting millions of patients worldwide, Yang et al. meta-analyze 22 randomized control trials finding that the use of CBT was associated with improved glycemic control. Importantly, certain characteristics of particular CBT interventions were associated with improved health outcomes; those interventions that had a shorter course of treatment <6 weeks (HbA1c decreased by 0.266; 95% CI of −0.328 to −0.204; *p* < 0.001), fewer than ten therapy sessions (HbA1c decreased by 0.276; 95% CI of −0.336 to −0.217, *p* < 0.001), lasting more than 90 min per session (HbA1c decreased by 0.274; 95% CI of −0.334 to −0.213, *p* < 0.001) were associated with improvement in glycemic control. The unique distinction here is that having a shorter, focused course of intervention may be more desirable to treat diabetes and perhaps other chronic diseases. One of the core foundations of CBT is the restructuring of internal beliefs. Targeting misconceptions about individual illnesses and, perhaps more importantly, allowing the patient to maintain a healthy self-concept in the midst of their chronic illness may help facilitate their recovery and aid in adherence with medical guidance. Similarly, addressing problematic behaviors associated with adherence is also a well-supported approach, further evidenced by the present findings. Importantly, while the disorders have difference nuances (e.g., medical/lifestyle interventions), the targets (i.e., cognition, behavioral patterns) are the same in which the clinician is attempting to modify the way the person perceives/interprets internal and external stimuli as well as corresponding maladaptive behaviors.

Complementing this, Pan et al. featured a Community Reinforcement Approach to CBT and found significant improvements in glycemic control in those with type-2 diabetes mellitus. Allowing patients with chronic conditions to connect with others who are struggling with the same health issues may be particularly therapeutic, especially as it inserts them into a network of peers that may provide models and tangible/emotional support regarding the management of their illness. Diabetes-specific CBT manuals have been compiled targeting improved diabetes management, understanding of the disease, and maladaptive behavioral patterns. Dismantling studies aimed at understanding optimal treatment components is highlighted as a needed next direction.

Several studies in this issue investigated innovative approaches to treating psychiatric and substance use concerns. Regarding early-onset schizophrenia, a study in this series by Li et al. found significant improvements in various cognitive domains (speed processing and attention/vigilance) when subjects were exposed to “Social Cognition and Interaction Training” (SCIT) in addition to paliperidone. Although the benefits of this specific training have not been studied over the long-term for this population, the hope would be that it and other psychological interventions (together with effective pharmacological management) may alter the trajectory of the disease by improving social relationships. Another condition in which a psychosocial intervention has been found beneficial is amphetamine-type stimulant use disorder (Tran et al.). This overview of systematic reviews with a supplemental meta-analysis found that various psychological interventions (e.g., contingency management, mindfulness, motivational interviewing, family therapy, etc.) caused persons to use injectables less frequently and helped prevent against engaging in risky sexual behavior. Interestingly, use of a contingency management strategy (i.e., a rewards system) helped improve treatment adherence among drug users, especially in those without severe mental illness. After the interventions were completed, however, the effects were found to diminish over time, implying the need for “booster” sessions or outright continuous psychological intervention.

Despite the availability of well-established interventions, there will always be room to explore new therapeutic modalities. Zhang et al. published a study protocol focused on mindfulness-based cognitive therapy for major depressive disorder to help clarify its potential role in treating that illness. This approach melds two evidence-based interventions to improve outcomes in a difficult to treat population. And Pchelina et al. proposed a study protocol focused on a novel internet-based CBT approach to treating insomnia. In addition to innovating new treatments, understanding mechanisms of action is important to get at the “why” a treatment works. Hagland et al. describes an association between the severity of obsessive-compulsive symptoms and delayed response rate during the “stop signal task,” providing a theoretical target for exposure and response prevention to enhance cognitive tasks. This is despite Hagland et al. not observing improvement in the “stop signal task,” after Bergen 4-Day Treatment format, suggesting the need to investigate the effect on the “stop signal task,” using other forms of CBT. Nevertheless, elucidating the mechanism of action for various psychosocial treatments will be an area of future research.

The role for psychotherapeutic interventions is robust, and the present issue highlights new innovations in treatment application and mechanisms. It will be important to investigate the way new modalities of psychotherapy can augment and perhaps even supplant current protocols of care. It may be crucial to dismantle components of CBT protocols to target disease-specific variations in response to treatment, an area of study requiring further clarification. With time we hope to see a more robust literature of various cognitive behavioral treatments that can target conditions previously thought divorced from needing psychotherapeutic intervention.

## Author Contributions

JB and ES were involved in developing and writing this editorial.

## Conflict of Interest

The authors declare that the research was conducted in the absence of any commercial or financial relationships that could be construed as a potential conflict of interest.

## Publisher's Note

All claims expressed in this article are solely those of the authors and do not necessarily represent those of their affiliated organizations, or those of the publisher, the editors and the reviewers. Any product that may be evaluated in this article, or claim that may be made by its manufacturer, is not guaranteed or endorsed by the publisher.

